# Polarization-independent achromatic Huygens’ metalens with large numerical aperture and broad bandwidth

**DOI:** 10.1515/nanoph-2023-0331

**Published:** 2023-08-18

**Authors:** Xiaoluo He, Chu Qi, Sheng Lei, Alex M. H. Wong

**Affiliations:** Department of Electrical Engineering, City University of Hong Kong, Hong Kong SAR, China; State Key Laboratory of Terahertz and Millimeter Waves, City University of Hong Kong, Hong Kong SAR, China

**Keywords:** achromatic metalens, Huygens’ metasurface, polarization-independent

## Abstract

Achromatic lenses, which have the same focal length regardless of the illumination frequency, find strong applications in imaging, sensing, and communication systems. Making achromatic lenses with metasurfaces is highly desirable because they are flat, ultrathin, relatively light, and easily fabricable. However, existing metalenses experience combinations of limitations which include single polarization operation, narrow bandwidth, and small numerical aperture (NA). In this work, we propose a dual polarized, broadband and high NA achromatic metalens based on the Huygens’ metasurface. We use Huygens’ metasurface unit cells with three tunable resonances to realize a stable group delay over a large bandwidth, while also achieving high transparency and large phase tunability. With these cells, we construct a dual-polarized achromatic Huygens’ metalens with an NA of 0.64 that works from 22 to 26 GHz. Our achromatic metalens achieves diffraction-limited focusing with 2 % maximum focal length deviation and 70 % average focusing efficiency over a bandwidth of 16.7 %. Most key performance metrics for this lens surpass or are comparable with the best of previous metalenses. An achromatic metalens simultaneously achieving broad bandwidth, large NA, and polarization-independent operation will open wide-ranging opportunities for microwave and mm-wave imaging and communication applications.

## 1 Introduction

The conventional refractive lens can focus or disperse light beams based on geometric optics and plays an irreplaceable role in many imaging systems. However, conventional lenses are generally bulky and suffer from aberrations and limited resolutions. These characteristics make them increasingly unsuitable for modern miniaturized photonic and electronic systems. The metasurface is an artificial planar structure composed of meta-atoms with unique electromagnetic properties and works effectively in manipulating wavefronts. Therefore, metasurface finds broad applications from microwaves to the optics [1–7], including anomalous reflection and refraction [1–3], beam generation [4, 5] and multifunctional optical devices [6, 7]. Among various kinds of metasurfaces, the metalenses specifically are designed for focusing and imaging systems, as they can flexibly control the amplitude, phase, and polarization of the incident light beams [8–10]. More importantly, metalenses can achieve some functions with a more effective design and lower cost than the conventional refractive lens, such as the aberration correlation, sub-diffraction-limited focusing, and wide-angle focusing [11–13].

One impediment to the practicality of a lens is its chromatic aberration, its shift in focal length as a function of frequency. This phenomenon distorts the signal and limits the bandwidth of many communication and imaging systems [14, 15]. The conventional optical system may use multiple optical components to correct for chromatic aberration, but this complicates the imaging system, increases its size, weight, cost, and reduces the focusing efficiency. Therefore, an achromatic metalens with subwavelength thickness and high focusing efficiency is highly desired.

Achromatic metalenses are first designed for optical imaging based on dielectric materials, working from infrared to the visible band [14–20]. The reported achromatic metalenses require close-packed meta-atoms [14–16] or meta-atoms with complex shapes [17, 19, 20] to provide the desired phase compensation and group delay. Moreover, some achromatic metalenses only work for circular-polarization waves as the design employs geometrical phase [14–16]. These features complicate the design and fabrication process and limit the focusing efficiency.

On the other hand, some effective metalenses have been demonstrated in the microwave region, which have expanded the practical applications of metalenses [21–28]. To correct the chromatic aberration, the frequency selective surfaces (FSSs) have been proposed based on the true time-delay filter synthesis theory [21, 28]. The advantage of FSSs is that the group delay can be well controlled by the number of dielectric layers [21, 28, 29]. However, these FSSs require several dielectric layers with different thicknesses to provide wide ranges of phase response and group delay, increasing challenges in the design and fabrication process. To achieve high transmission efficiency with ultrathin thickness, the Huygens’ metasurfaces have been proposed, as they are able to achieve broad phase ranges by locating orthogonal equivalent electric and magnetic currents with a subwavelength thickness [30, 31]. Several Huygens’ metalenses have been proposed and applied for antenna gain enhancement [22, 23] and mm-wave focusing [26, 27]. Nevertheless, the proposed Huygens’ metalenses have either limited focusing bandwidths or small numerical apertures (NAs), which may limit their applications. Enlarging the effective NA and broadening the bandwidth of a metalens is crucially important at mm-wave frequencies, contributing to various applications, including wireless communication, imaging, and sensing.

**Figure 1:**
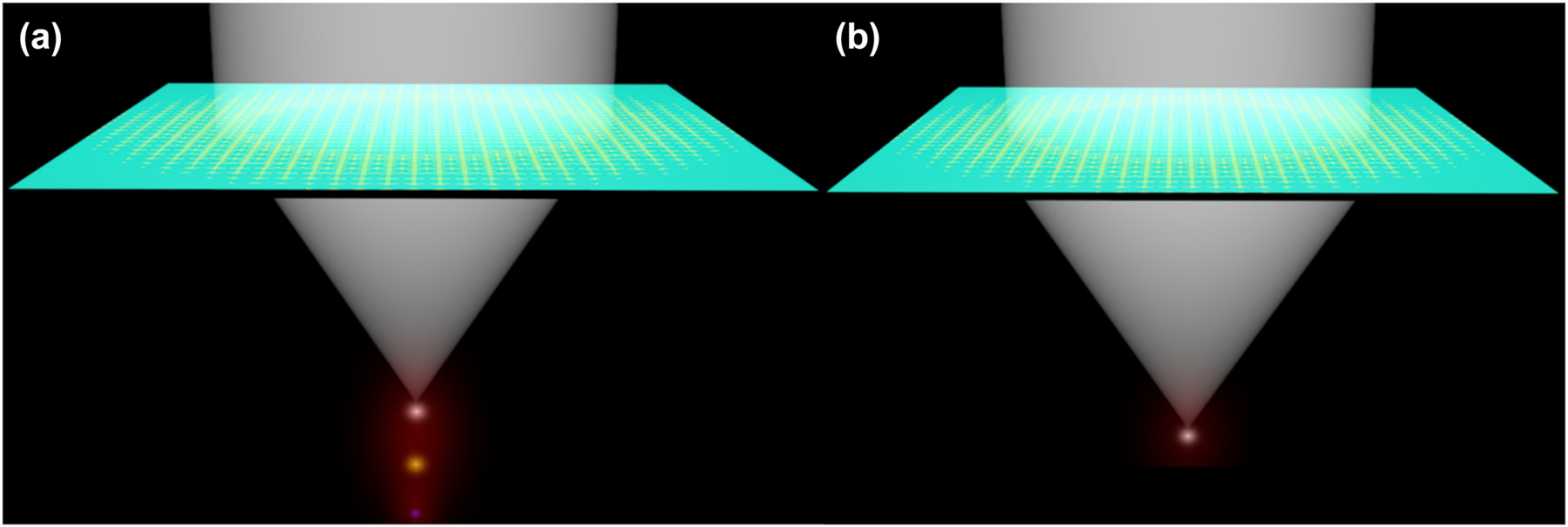
The schematic diagrams of metalenses. (a) The chromatic metalens (the focal length shifts with the change of incident wavelength). (b) The achromatic metalens (the focal length is constant with the change of incident wavelength).

In this paper, we propose a polarization-independent achromatic metalens with large NA and broad bandwidth operation. A schematic of the achromatic metalens is shown in Figure 1. We construct the metalens using a multiresonant Huygens’ unit cell with two electric and one magnetic resonances. Proper unit cell design enables us to independently tune all three resonances, which allows us to control the group delay over a broad bandwidth, and at the same time maintain high transparency and full 2*π* phase tunability. These traits enable us to design achromatic lenses with large bandwidths and large numerical apertures [11, 32]. Thereafter, we use these unit cells to realize a polarization-independent achromatic metalens in the K-band (22–26 GHz) with an NA of 0.64. The achromatic metalens achieves diffraction-limited focusing with a stable (±1 %) focal length, and a high focusing efficiency (70 % on average) over the operation band. The achromatic focusing bandwidth reaches 16.7 %, which is close to the theoretical bandwidth limit for passive and lossless metalenses [32, 33]. The simultaneous achievement of polarization independence, large NA and broad achromatic bandwidth makes our metalens attractive for various microwave and mm-wave lensing applications.

## 2 Theory and structure

In this section, we first introduce and briefly overview the Huygens’ metasurface. Then, we realize a set of Huygens’ metasurface unit cells with high transmission efficiency and broad phase tuning range by designing the admittances of three metallic layers. These admittance sheets give us independent control of electric and magnetic resonances. By tuning the number of resonances and their positions, we achieve broad-ranged tuning of phase response and group delay of unit cells. Lastly, using the designed unit cells, we develop an achromatic Huygens’ metalens showcasing the effectiveness of our method. While we reported preliminary simulation results in Ref. [34], this paper constitutes a full report of our work, including theory, simulation, experiment, and a comparison with relevant previous works.

### 2.1 Huygens’ metasurfaces

**Figure 2:**
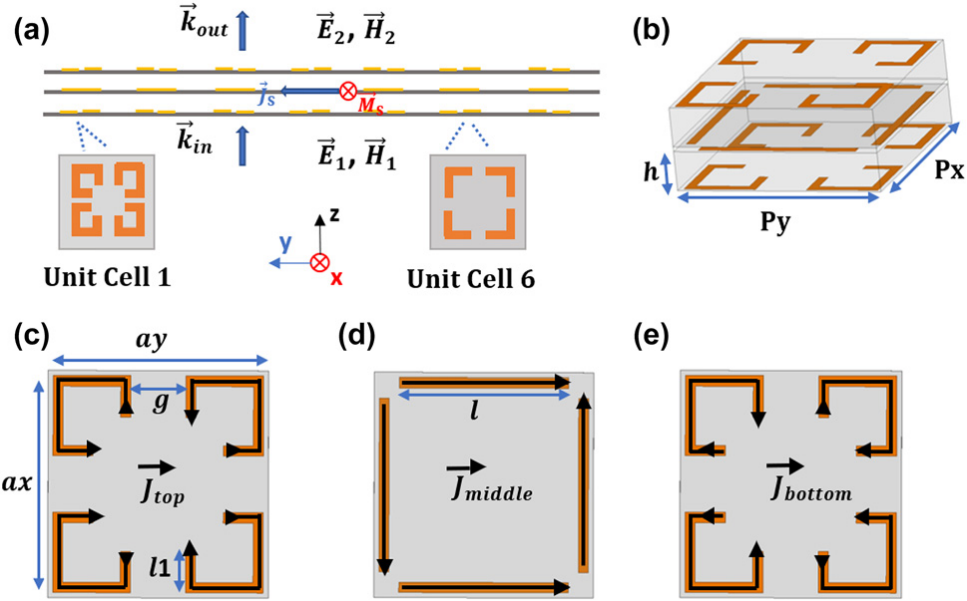
The structure of designed dual-polarized metasurface unit cell. (a) The schematic diagram of designed HMS (cross-section view). (b) The schematic diagram of the unit cell. (c–e) The details of three metallic layers (top-view): (c) the top layer, (d) the middle layer, (e) the bottom layer. The brown lines indicate metallic traces, the black arrows on the metallic traces indicate the direction of current travel at one phase point.

We begin with a dual-polarized unit cell design based on the principle of Huygens’ metasurfaces (HMSs) [30, 35], which has advantages of miniaturized size, broad phase response, and wide group delay range. In general, an HMS is composed of a thin surface with co-located and orthogonal electric and magnetic dipoles. When discontinuous fields ( $\vec{E_{1}},\vec{H_{1}}$ and $\vec{E_{2}},\vec{H_{2}}$ ) in two half-space are separated by the HMS, $\left(\vec{J_{s}}\right)$ and magnetic $\left(\vec{M_{s}}\right)$ current densities can be induced to transform the fields from one half-space to the other half-space, as shown in Figure 2(a). From the boundary conditions, the fields on both half regions can be expressed as [30, 35]:

(1) $$\begin{array}{c} \vec{J_{s}}=\hat{n}\times \left(\vec{H_{2}^{+}}-\vec{H_{1}^{-}}\right)={\bar{\bar{Y}}}_{\text{es}}\cdot \vec{E_{\text{av}}^{t}}{|}_{s}\\ \vec{M_{s}}=\hat{n}\times \left(\vec{E_{2}^{+}}-\vec{E_{1}^{-}}\right)={\bar{\bar{Z}}}_{\text{ms}}\cdot \vec{H_{\text{av}}^{t}}{|}_{s} \end{array}$$

where ${\bar{\bar{Y}}}_{\text{es}}$ and ${\bar{\bar{Z}}}_{\text{ms}}$ represent the electric surface admittance and magnetic surface impedance, respectively. $\vec{E_{\text{av}}^{t}}{|}_{s}$ and $\vec{H_{\text{av}}^{t}}{|}_{s}$ are the average tangential fields of the surface. Generally, the surface admittance $\left({\bar{\bar{Y}}}_{\text{es}}\right)$ and impedance $\left({\bar{\bar{Z}}}_{\text{ms}}\right)$ are tensors. However, they can be simplified to scalar quantities (*Y*_es_ and *Z*_ms_) for a single polarization surface or polarization-insensitive surfaces [23, 31, 35]. Hereafter we adopt this simplification. These impedance and admittance values can be designed with spatially sampled subwavelength unit cells. According to the boundary condition and the equivalence principle [30, 35], the normalized admittance *Y* and impedance *Z* can be extracted from the complex coefficients of reflection (*r*) and transmission (*t*):

(2) $$\begin{array}{c} Y=Y_{\text{es}}\eta_{0}=\frac{2\left(1-t-r\right)}{\left(1+t+r\right)}\\ Z=\frac{Z_{\text{ms}}}{\eta_{0}}=\frac{2\left(1-t+r\right)}{\left(1+t-r\right)} \end{array}$$

where *η*_0_ is the wave impedance of free space. When Re $\left\{Y\right\}$ = Re $\left\{Z\right\}=0$ and Im $\left\{Y\right\}$ = Im $\left\{Z\right\}$ , Eq. (2) requires that the surface is reflectionless (*r* = 0) and a broad transmission phase variation with respect to frequency can be realized [3, 30]. Based on the reflectionless property of the HMS, many interesting and efficient metadevices have been demonstrated for beam steering [36–38], beam generating [39], and beam focusing [22, 23].

### 2.2 Unit cell design

We begin with a very brief overview on existing HMSs most relevant to broadband achromatic lensing, their capabilities and their limitations. We then list important properties of a unit cell which will lead to a broadband achromatic metalens. Although existing HMSs show excellent conversion efficiency between the incident and refracted waves with deeply subwavelength thickness, the reported works mostly focus on resonance matching under a single frequency, resulting in limited working bandwidths [22, 23, 36, 37, 39]. Recent works show that broadband HMSs can be realized if the resonances between three metallic layers are controlled independently [24, 27]. However, Ref. [24] ignored phase dispersion within the bandwidth, resulting in a strong focal length shift, while Ref. [27] has a limited phase and group delay coverage, as it only engineers one electric and one magnetic resonance around the operating bandwidth. To construct a highly desirable achromatic metalens with large NA and broad bandwidth, the unit cells should meet the following requirements: (i) high transmission efficiency, (ii) broadly tunable transmission phase at the center frequency, (iii) linear phase response, and (iv) broadly controllable group delay. Here, we propose a dual-polarized Huygens’ unit cell with three metallic layers, which can simultaneously achieve these requirements by engineering two electric and one magnetic resonances within or near the operating bandwidth.

Figure 2 depicts our unit cell structure. Its symmetric pattern distribution ensures polarization insensitivity for *x*- and *y*- polarized waves. The outer layers (top and bottom layers) contain four corner strips which vary in length, and in some cases become folded (see Figure 2(c)–(e)). The middle layer contains four straight strips along the four sides of the cell. Although this unit cell is simple, we will show that it provides sufficient control over two electric resonances and one magnetic resonance. Specifically, the outer layer controls the magnetic resonance and one of the electric resonances: the magnetic resonance originates from the antisymmetric surface currents between the outer layers, while the electric resonance originates from the symmetric surface currents in the same layers. The other electric resonance is generated by the four strips on the middle layer. We can tune these three resonances by sweeping three variables: the gap between corner strips of outer layers (*g*), the folded strip length of outer layers (*l*_1_) and the length of four strips in the middle layer (*l*).

To demonstrate that the magnetic resonance and electric resonances can be tuned independently, we analyze the unit cell with the surface current method [39]. Assuming the unit cell is illuminated with a y-polarized EM wave, the induced surface currents ${\vec{J}}_{\text{top}}$ , ${\vec{J}}_{\text{middle}}$ and ${\vec{J}}_{\text{bottom}}$ are formed on the top, middle, and bottom layers, respectively. The arrows on the strips indicate the travel direction of currents ${\vec{J}}_{\text{top}}$ , ${\vec{J}}_{\text{middle}}$ and ${\vec{J}}_{\text{bottom}}$ at the same time instance, as shown in Figure 2(c)–(e). The propagation distance separating the top and bottom layers, along with coupling dynamics, make ${\vec{J}}_{\text{top}}$ and ${\vec{J}}_{\text{bottom}}$ generally unequal [39]. ${\vec{J}}_{\text{top}}$ and ${\vec{J}}_{\text{bottom}}$ can be decomposed into the common and differential modes, which give rise to electric and magnetic responses respectively. The induced current of the middle layer mainly comes from the four capacitors, formed by the pairs of metallic strips on the four sides of the unit cell. Thus the surface currents on each layer can be decomposed as:

(3) $$\begin{array}{cc} {\vec{J}}_{\text{top}} & ={\vec{J}}_{\text{com}}+{\vec{J}}_{\text{diff}},\\ {\vec{J}}_{\text{bottom}} & ={\vec{J}}_{\text{com}}-{\vec{J}}_{\text{diff}},\\ {\vec{J}}_{\text{mid}} & ={\vec{J}}_{\text{mid}} \end{array}$$

${\vec{J}}_{\text{com}}$ and ${\vec{J}}_{\text{diff}}$ represent the currents of common and differential modes, respectively. As the unit cell is symmetric with respect to the middle layer, the current ${\vec{J}}_{\text{mid}}$ couples equally to the top and bottom layers. Thus this set of coupled currents contribute to the common mode, but not the differential mode. The converse — that ${\vec{J}}_{\text{com}}$ would contribute a coupled current to the middle layer, but ${\vec{J}}_{\text{diff}}$ would not — is true by reciprocity. To summarize, (i) the magnetic dipole is arises solely from ${\vec{J}}_{\text{diff}}$ , which can be tuned by adjusting the outer layers; (ii) the two electric responses are predominantly affected by ${\vec{J}}_{\text{com}}$ (tuned by adjusting the outer layers) and ${\vec{J}}_{\text{mid}}$ (tuned by adjusting the middle layer) respectively; and (iii) the two electric dipoles will mutual couple with one another. Based on these observations, an efficient way to design the broadband unit cell is to first tune the outer layers to obtain a desirable magnetic response, then adjust the parameters of the middle layer to obtain the desired composite electric response, and thereby achieve the desired transmission coefficient and broadband group delay. The relationship between resonances and variables can be found in Supplementary Note S1.

**Figure 3:**
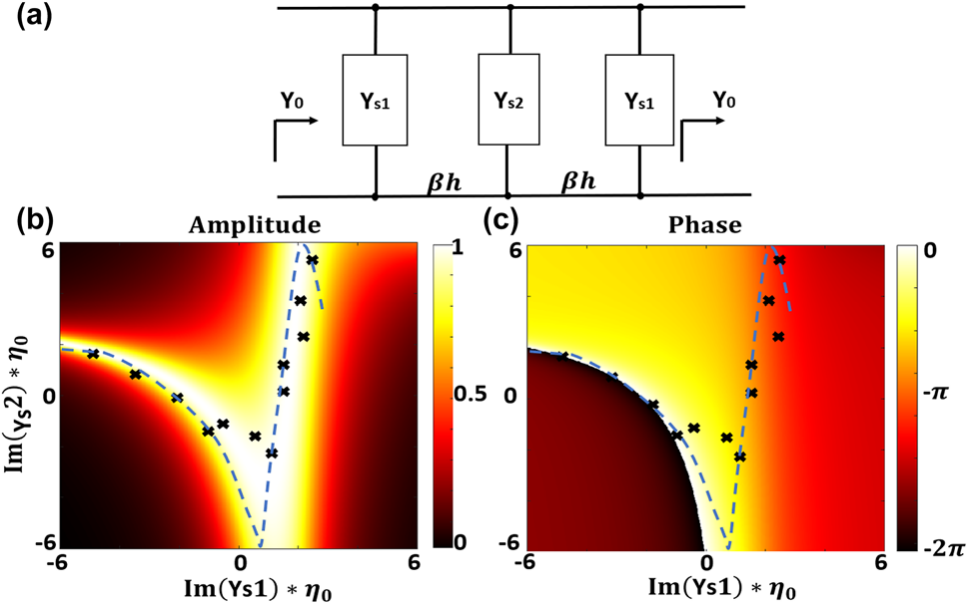
Circuit modelling of the metasurface unit cell. (a) The equivalent circuit model of cascaded three-layer metasurface unit cell. (b) The transmission amplitude as a function of *Y*_*s*1_ and *Y*_*s*2_. (c) The transmission phase as a function of *Y*_*s*1_ and *Y*_*s*2_.

The equivalent circuit model of our unit cell structure is presented in Figure 3(a). *β* and *h* represent the wavenumber and the thickness of the substrate, and *Y*_*s*1_ and *Y*_*s*2_ are the equivalent admittances of the outer and middle layers, respectively. Once the input and load admittances, substrate parameters, and reflection and transmission coefficients are specified, the required admittances of different sheets can be calculated by the ABCD matrix method (see Supplementary Note S2). Figure 3(b) and (c) show the transmission amplitude and phase achievable by tuning *Y*_*s*1_ and *Y*_*s*2_ at the center frequency of 24 GHz. Particularly, by tuning (*Y*_*s*1_ and *Y*_*s*2_) along the dashed blue lines in Figure 3(b) and (c), the transmission amplitude can remain close to unity while the phase varies by more than 360°. This shows that the three-layer isotropic Huygens’ metasurface topology can yield high transmission efficiency and over 360° phase range, provided that *Y*_*s*1_ and *Y*_*s*2_ can be tuned over the depicted range. The black “*x*”s in Figure 3(b) and (c) depict the (*Y*_*s*1_) and (*Y*_*s*2_) values we achieved with our metalens unit cells. This shows that our proposed unit cell structure indeed provides sufficient tuning range to achieve near-unity transmission and over 360° phase coverage. We now examine how the ability to flexibly tune *Y*_*s*1_ and *Y*_*s*2_ leads to desirable broadband characteristics for the metasurface unit cell. Refs. [27, 37] have shown that *Y*_*s*1_ and *Y*_*s*2_ completely determine the surface admittance and impedance *Y*_es_ and *Z*_ms_ of the metasurface unit cell. The independent control of *Z*_ms_ and *Y*_es_ allows us to leverage the “Huygens” characteristics of the unit cell and achieve high transmission efficiency over a broad bandwidth. We will also show, by way of example, that by positioning the resonances we can also achieve controllable linear phase changes during the entire operating bandwidth.

**Figure 4:**
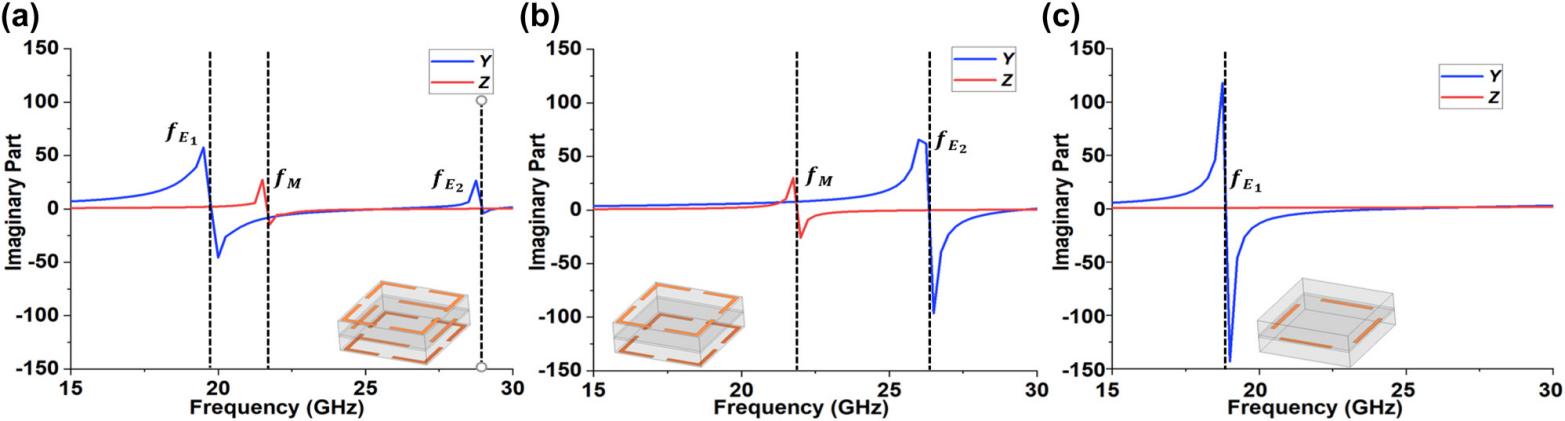
Imaginary parts of normalized admittance *Y* and impedance *Z* of the unit cell 3. (a) The unit cell with three layers. (b) The unit cell with only the end layers. (c) The unit cell with only the middle layer.

We simulate the electromagnetic response of the proposed unit cell using Ansys HFSS. We use a Rogers RO4003C dielectric substrate with a relative permittivity of 3.55 and a thickness of 0.813 mm. The patterned copper has a thickness of 9 um and a conductivity of 5.8 × 10^7^ Ω^−1^ m^−1^. In our design, the length of the corner strip on the outer layers is *a*_*x*_ = *a*_*y*_ = 4.2 mm and the unit cell size is *P*_*x*_ = *P*_*y*_ = 4.4 mm. A periodic boundary is used in the *x*- and *y*- directions to simulate an infinite 2D array of identical cells. Figure 4 gives the imaginary parts of the normalized electric admittance (*Y*) and magnetic impedance (*Z*) versus frequency of one unit cell used in our achromatic metalens. The values of *Y* and *Z* can be retrieved by incorporating the transmission and reflection coefficients into Eq. (2). In this example, the three variables are *g* = 0.4 mm, *l* = 3.3 mm, and *l*_1_ = 0.2 mm. We find that the unit cell has two electric resonances (at $f_{E_{1}}$ and $f_{E_{2}}$ ) and one magnetic resonance (at *f*_*M*_), as shown in Figure 4(a). To analyze the electric and magnetic responses, we separate the three metallic patterns into the outer layers and the middle layer, as shown in Figure 4(b) and (c). The outer layers support one magnetic resonance (*f*_*M*_) and one electric resonance $\left(f_{E_{2}}\right)$ , while the middle layer only supports one electric resonance $\left(f_{E_{1}}\right)$ . When we combine the middle and outer layers, we find that the magnetic resonance response (*f*_*M*_) remains unshifted, but the two electric resonances ( $f_{E_{1}}$ and $f_{E_{2}}$ ) are shifted because of mutual couplings among the three layers. The forgoing observations show that the outer layers mainly control the magnetic response while all three layers and their coupling dynamics affect the electric response, which is consistent with previous works reported in Refs. [27, 37, 39], and our insights obtained through the surface current perspective earlier this section.

**Figure 5:**
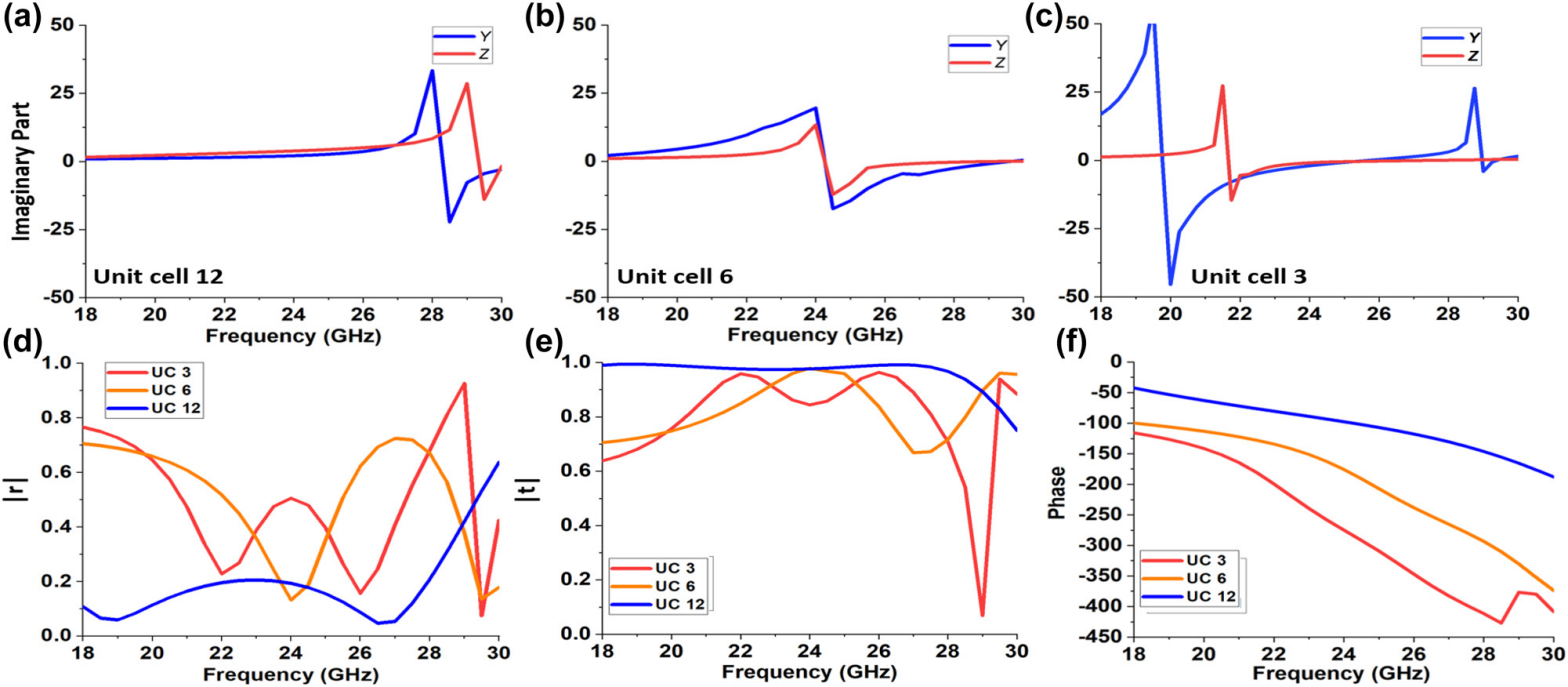
Schematic diagrams of three unit cells. (a–c) Imaginary parts of normalized admittances *Y* and impedances *Z* of unit cell 12 (a), unit cell 6 (b) and unit cell 3 (c). (d) Reflection coefficients of three unit cells. (e) Transmission coefficients of three unit cells. (f) Transmission phases of three unit cells.

A broad range of group delay and near-zero group delay dispersion within the bandwidth is the key to achieving achromatic metalens with large NA [14, 32]. In this work, we achieve a linear phase variation and broadly controllable group delay by independently controlling two electric and one magnetic resonances. In general, around the resonance frequency, the transmission coefficient changes rapidly in phase and decreases somewhat in magnitude. Making use of this effect, one can achieve a linear phase with controllable dispersion by tuning the spectral positions of resonances respect to the operating bandwidth. We shall illustrate this using examples of three unit cells from our metalens, for which the surface magnetic impedances and surface electric admittances are shown in Figure 5(a)–(c). Unit Cell 12 has two resonances located away from the operation band (22–26 GHz). Correspondingly, Figure 5(e) and (f) (blue, solid) show that this unit cell achieves near-unity transmission amplitude and a slow phase variation over the operation band (i.e. a small group delay). Unit Cell 6 (Figure 5(b), (e), and (f)) achieves a faster phase variation (or a larger group delay) by having two resonances inside the operating band. Finally, Unit Cell 3 (Figure 5(c), (e), and (f)) achieves an even faster phase variation by involving three resonances positioned just outside the operation band. Thus by tuning spectral positions of the resonances, the group delay can be kept mostly constant over the operation band and its slope can be tuned. While the usage of multiple resonances close to, or within, the operation band may reduce the transmission efficiency, we find that even for such cases, absorption and reflection can be kept minimal by using a weakly resonant (low-Q) unit cell design and keeping a similar normalized *Y* and *Z* [40,41] . It should be noted that the transmission coefficient reaches its peak when Im $\left\{Y\right\}$ = Im $\left\{Z\right\}$ . The frequency of maximum transmission corresponds to the resonant frequency of the complete unit cell, which in general differs from the frequencies of the three individual resonances that make up the cell. Figure 5(d) and (e) show that for all three unit cells, high transmission efficiencies are achieved at the design frequency of 24 GHz, and a transmission amplitude of >0.8 is achieved over the operation band. In summary, we find that our proposed unit cell provides flexible tunability for all three resonances, which is sufficient to design metasurface unit cells with high transmission efficiency, linear phase variation, and broadly controllable group delay over a wide bandwidth. Hence they are attractive for the construction of the high-transmission-efficiency, broadband achromatic metalens reported in this paper. The resonances performance of all unit cells can be found in Supplementary Figure S3.

### 2.3 Achromatic metalens design

To achieve achromatic focusing, the spatial distribution of unit cells should simultaneously perform phase and group delay compensations. Firstly, the phase compensation of the unit cell according to the spatial location can be expressed by the equal wave travel distance as:

(4) $$\phi \left(r,\omega \right)=-\frac{\omega }{c}\left(\sqrt{F^{2}+r^{2}}-F\right)$$

where *ω*, *c*, *F* and *r* represent the angular frequency, speed of light, focal length, and radial coordinate of the unit cell, respectively. Moreover, for the achromatic metalens, the required group delay, as a functional of the radial position, can be expressed as:

(5) $$\Delta TD\left(r\right)=\frac{\sqrt{R^{2}+F^{2}}-\sqrt{r^{2}+F^{2}}}{c}$$

where Δ*TD*(*r*) is the relative group delay and *R* is the radius of the metalens. We find that the greatest delay is required at the center of the metalens (i.e. *r* = 0). A large range of relative group delay is required when the NA of the achromatic lens is large, as the NA can be defined as:

(6) $$NA=\frac{\text{R}}{\sqrt{{\text{R}}^{2}+{\text{F}}^{2}}}$$

**Figure 6:**
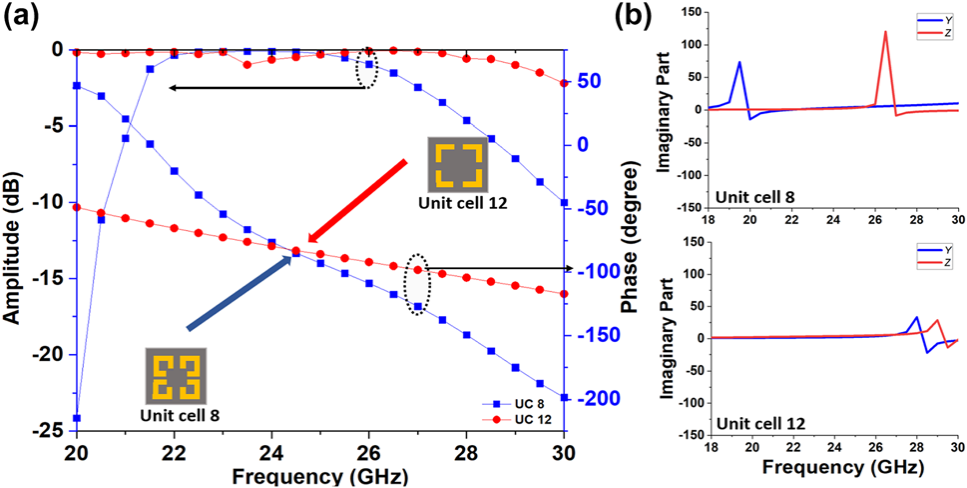
Comparison of two constructed unit cells. (a) The transmission coefficients of two unit cells. (b) Imaginary parts of normalized admittance and impedance of unit cell 8 and 12 respect to the frequency.

A comparison of required phase distributions and relative group delays of two achromatic metalenses with different NAs is provided in Supplementary Figure S4. It shows that to construct an achromatic metalens with a large NA, unit cells should have a transmission phase coverage of more than 360° and a broad range of group delay. Specifically, some unit cells of the achromatic metalens should have the similar phase response at the center frequency but with different group delays. Most proposed achromatic metalens fail to achieve that, leading to designs with small NAs [14], [15], [16, 18, 21, 27]. In our case, we achieve the required conditions through the independent control of multiple resonances. Figure 6(a) gives the transmission coefficients of two unit cells, which illustrate this point. Both unit cells have high transmission amplitudes over the operating frequency (22–26 GHz) and the same phase response under the center frequency (24 GHz), but they have very different group delays. Figure 6(b) and (c) give the explanation, where for Unit cell 8, the electric and magnetic resonances are closer to the operating bandwidth, resulting in a narrower fractional bandwidth compared to Unit cell 12. Hence by introducing the three-layer Huygens’ metasurface unit cell, and properly tuning the resonances it affords, we can construct a library of unit cells with high transmission efficiency, large phase range (at 24 GHz) and large group delay range, which can then be used to construct a high-performance achromatic metalens.

**Figure 7:**
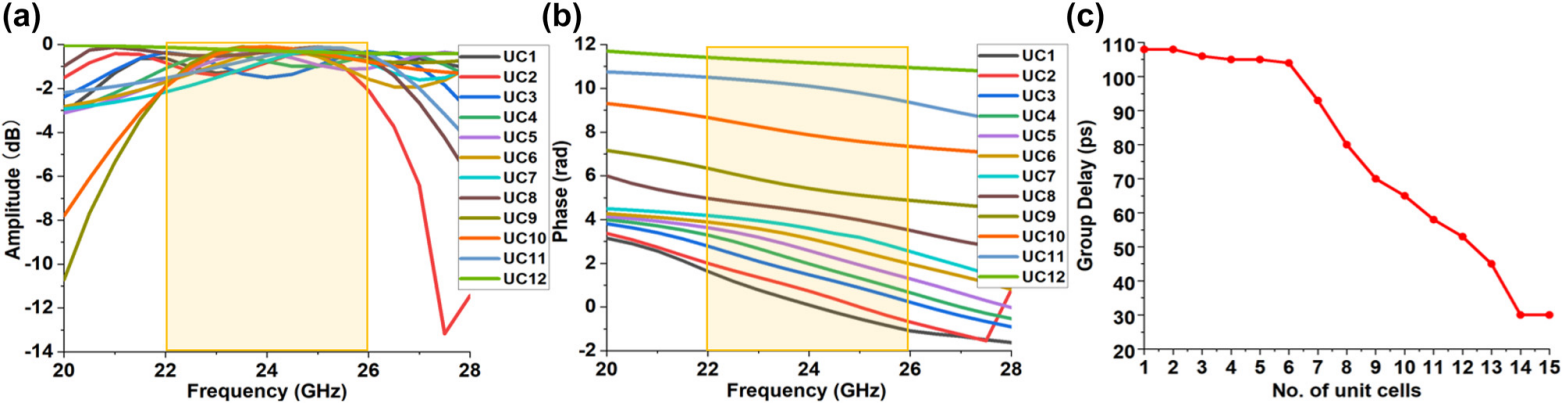
Transmission coefficients of the unit cells of the achromatic metalens (the yellow region is the bandwidth of interest). (a) The transmission amplitude of the used unit cells. (b) The transmission phase response of the used unit cells. (c) The group delay distribution of the used unit cells.

Based on the unit cell library, we design a polarization-independent achromatic metalens with NA equals to 0.64 by using 12 different unit cells. The transmission amplitudes and phases of these unit cells are given in Figure 7(a) and (b), respectively. It can be found all unit cells can achieve larger than −2 dB transmission amplitude over the operation bandwidth from 22 GHz to 26 GHz, which enables high transmission and focusing efficiencies of our metalens. The group delays of unit cells from the center to the edge gradually decrease, as seen in Figure 7(c). This ensures effective chromatic correction of our metalens. The geometrical parameters and corresponding time delay of unit cells are given in Supplementary Table S1.

**Figure 8:**
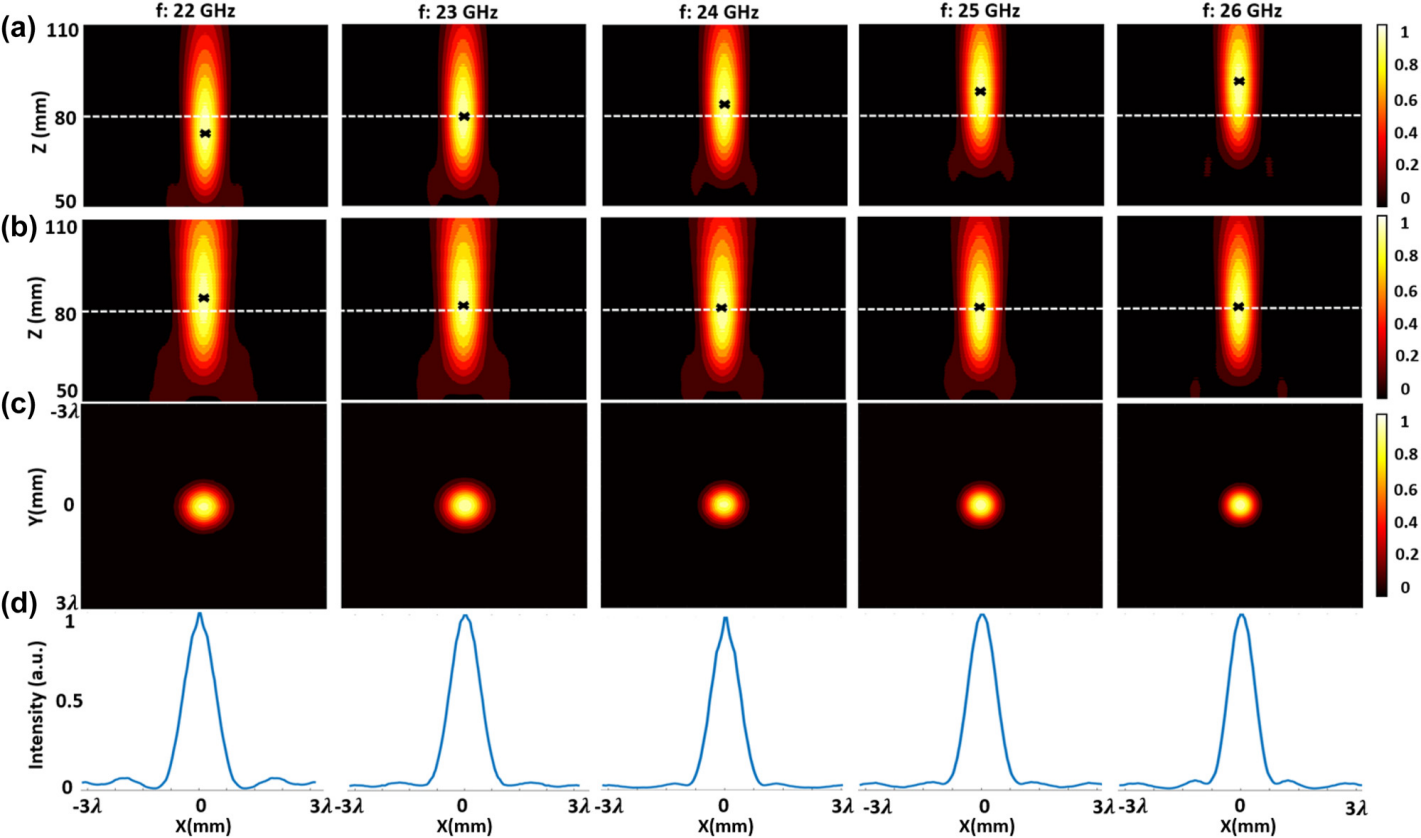
Simulated field distributions of the chromatic and achromatic metalenses. (a) Normalized intensity distribution of chromatic metalens along the propagation plane (*XZ*-plane). (b) Normalized intensity distribution of achromatic metalens along the propagation plane (*XZ*-plane). (c) Normalized intensity distribution along the focal plane (*XY*-plane, 2D). (d) Normalized intensity distribution curves in the *x*-direction crossing the center of each focal hot spot. (*λ* = 12.5 mm is the free-space wavelength of 24 GHz EM wave.)

We carry out a full-wave simulation of the achromatic metalens using Ansys HFSS. To verify the performance of the achromatic metalens, we simulated the chromatic metalens and the achromatic metalens. Both metalenses are designed with the same aperture size and focal length, by using the designed unit cell structure but with different group delay distributions. Theoretically, the chromatic metalens should have constant group delay for all unit cells. However, in our case, the unit cells used for the chromatic metalens have a small variation of group delay as the phase is tuned by the resonance. Figure 8 gives the normalized intensity profiles at the *XZ*-plane for both metalenses and the focal plane for achromatic metalens under different frequencies. The “*x*” represents the designed focal spot. The focal length has shifted slightly from 80 mm for both metalenses. From Figure 8(a) and (b), it can be seen that the focal length of the chromatic metalens increases from 76 mm to 93 mm with the increase of frequency, while the focal length of the achromatic metalens is at 83 ± 1.5 mm over the whole bandwidth. In addition, the chromatic aberration of a conventional metalens can be described as [42]:

(7) $$\Delta F=F\frac{\Delta \lambda }{\lambda }$$

where *λ* and *F* are the central wavelength and designed focal length of the metalens, respectively. Δ*F* represents the corresponding focal shift within the bandwidth. From Eq. (7), we can find that the achromatic metalens has improved the focal length shift from 13.44 mm (in theory) to 1.5 mm (in simulation), achieving an 89 % improvement. Figure 8(c) and (d) show the normalized intensity distributions on the focal plane. We find that the achromatic metalens has a very low sidelobe level (less than 5 % of the peak) within the working bandwidth. The corresponding full-width half maximum (FWHM) of the spot size is close to the diffraction-limited $\text{FWHM}=\frac{0.5\lambda }{\text{NA}}$ . In addition, the metalens has achieved a very high transmission efficiency of 90 %, and a maximum focusing efficiency of 80 % (at 23 GHz). The transmission efficiency is the fraction of power transmitted by the metasurface [24, 27]. The focusing efficiency is defined as the ratio between the integral of the field intensity in the focal plane with a diameter six times the FWHM spot size, to the full plane integral of field intensity in the focal plane [26, 27, 43]. We also simulate the focusing performance of the achromatic lens under oblique incidence and find that the focal length can remain constant over the whole bandwidth upon a moderate oblique incidence of up to 30°. (See Supplementary Note S6 and Supplementary Figures S6 and S7).

## 3 Experimental demonstration

**Figure 9:**
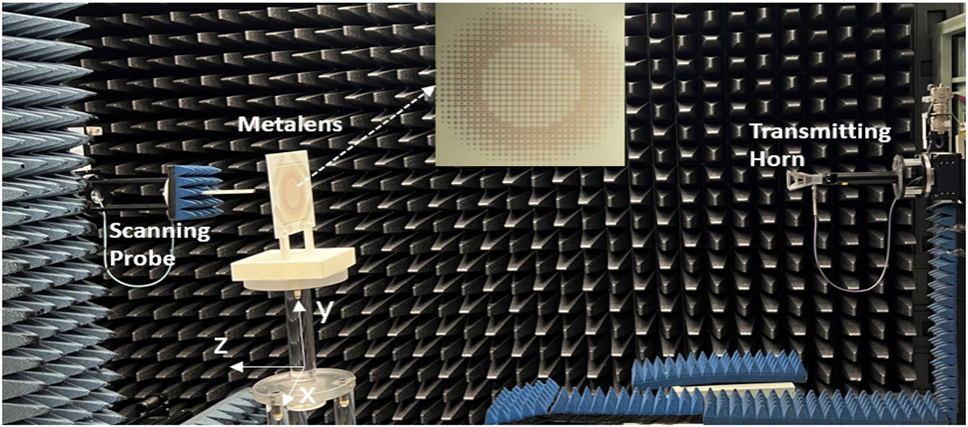
The schematic of experimental set up.

We proceed to fabricate and characterize the proposed achromatic metalens. Figure 9 shows the setup for our experiment and gives the detail of the achromatic metalens. The size of the fabricated metasurface is 132 × 132 mm^2^. A transmitting antenna (a 4–40 GHz double ridged horn NSI-RF-RGP-40) is placed far enough from the achromatic metalens to launch an incident wave with a reasonably planar wavefront. In the experiment, the metasurface is embedded into a 3D-printed holder, and the central point of the metalens is defined as the origin (*x* = *y* = *z* = 0). A probe is used to scan the electrical field distribution of the *XY*-plane from *z* = 50 mm to *z* = 110 mm.

**Figure 10:**
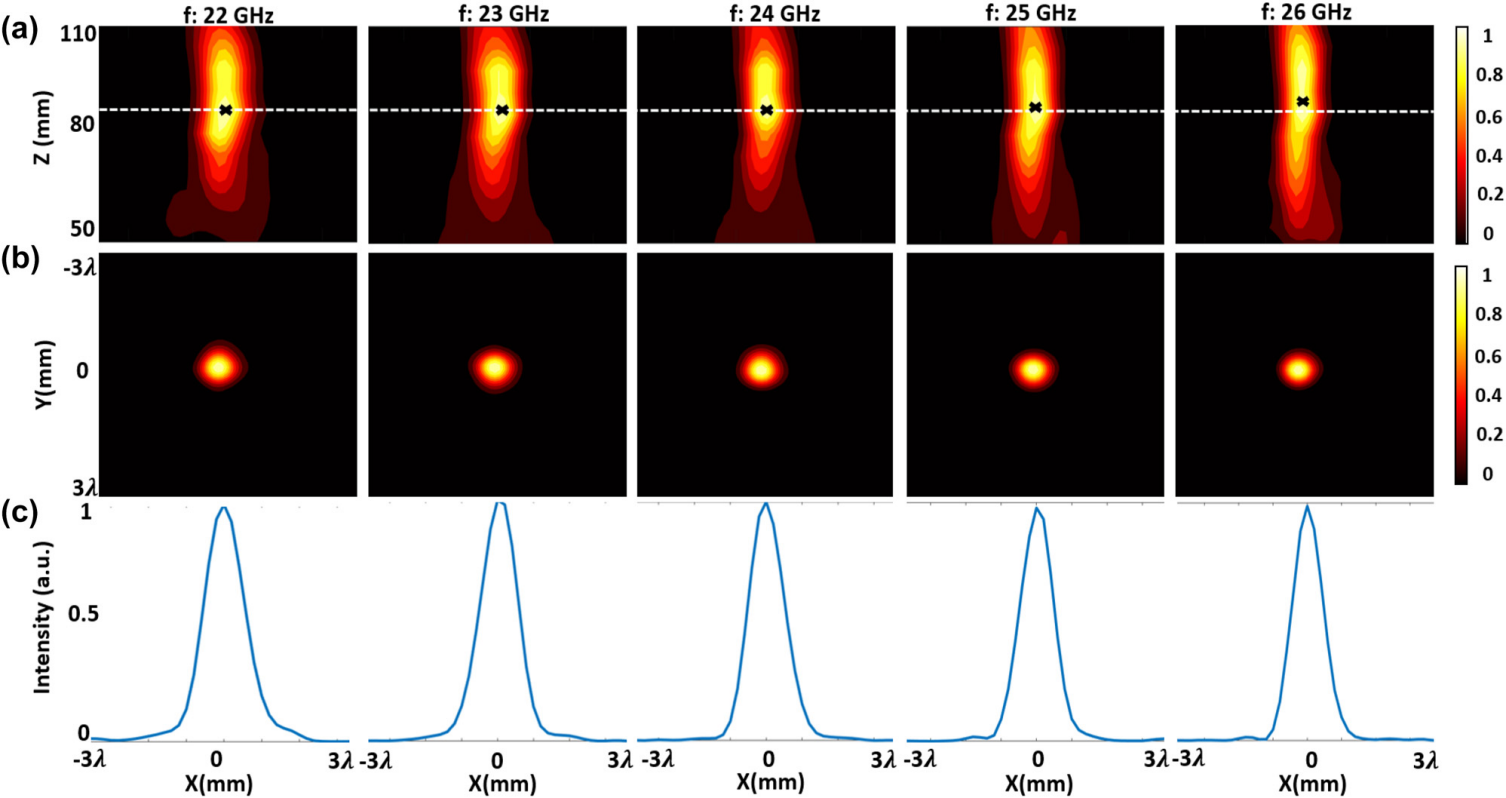
The measured field distribution of the achromatic metalens. (a) Normalized intensity distribution along the propagation plane (*XZ*-plane). (b) Normalized intensity distribution along the focal plane (*XY*-plane, 2D). (c) Normalized intensity distribution curves in the *x*-direction crossing the center of each focal hot spot. (*λ* = 12.5 mm is the free-space wavelength of 24 GHz EM wave.)

**Figure 11:**
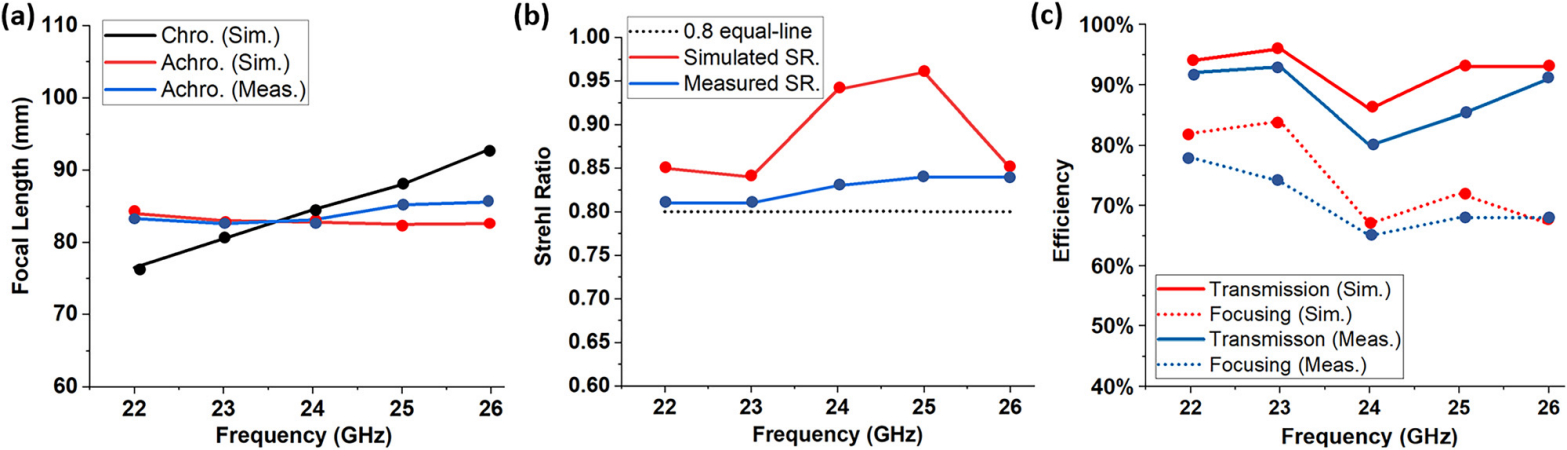
Comparison between simulated and measured results of (a) focal length. (sim.: simulated results, meas.: measured results). (b) Calculated Strehl ratio. (SR.: Strehl ratio). (c) The transmission and focusing efficiency.

Figure 10 gives the measured field distributions of different planes over the bandwidth of interest. Figure 10(a) shows the focal spot in the *XZ*-plane for a range of frequencies within the operation band of 22–26 GHz. The focal spot shifts slightly to the left for distance around *z* = 75 mm, due to a slight lateral misalignment in the measurement setup. Nevertheless, this does not preclude the main observations of the experiment. We find that the fabricated achromatic metalens has a good focusing performance over the frequency range of interest, and the focal length versus frequencies remains almost unshifted across the operation band. Figure 10(b) and (c) give the normalized intensity distributions of the focal plane (*XY*-plane). The spot sizes (FWHM) under different frequencies are around 0.88*λ*, which are close to the diffraction limit of 0.78*λ* (for NA = 0.64) and are consistent with the simulated results. This shows the broad focusing bandwidth of our achromatic metalens. Figure 11(a) compares the measured focal length of the achromatic metalens with the simulated results for chromatic and achromatic metalenses. The Strehl ratios under different frequencies are calculated by comparing the simulated or measured intensity distribution of the focal spot to the theoretical intensity distribution with $\text{FWHM}=\frac{0.5\lambda }{\text{NA}}$ [14]. A metalens can be regarded as diffraction-limited if the Strehl Ratio is larger than 0.8. Figure 11(b) compares the simulated and measured Strehl Ratio, which shows the achromatic metalens is indeed diffraction-limited over the working bandwidth. The simulated and measured transmission efficiencies and focusing efficiencies are given in Figure 11(c). The transmission efficiency is over 80 % within the entire bandwidth due to the high transmission efficiency of unit cells, and the average focusing efficiency is at 72 % from 22 GHz to 26 GHz, corresponding to a fractional bandwidth of 16.7 %. The experimental results are in good agreement with simulation results and indicate our metalens has achieved power efficient, low sidelobe achromatic focusing over the working bandwidth.

Table 1 compares our achromatic metalens with the most relevant previous works. In this comparison, we distinguish two bandwidths — one for the 3 dB focusing efficiency and the other for achieving diffraction-limited focusing with a near-constant focal length. For ease of distinction, we call the former the 3 dB bandwidth and the latter the achromatic bandwidth. The 3 dB bandwidth is defined as the frequency range in which the focusing efficiency has dropped to half of its peak value [24, 25]. For our metalens, the simulated 3 dB bandwidth stretches from 21 GHz to 30 GHz, while the experimental bandwidth is slightly lower, from 22 GHz to 30 GHz (see Supplementary Note S7 and Figures S8 and S9). This means our metalens has an experimental fractional 3 dB bandwidth of 30.7 %, which is wider than or comparable to previously proposed chromatic metalenses. In terms of achromatic lensing, the achromatic lensing bandwidth is comparable to previous works, outperforming [27] but inferior to Ref. [28]. However, within the operation bandwidth, our metalens reduces chromatic aberration by 89 % while [28] reduces chromatic aberration by only 75 %. Hence while our claimed achromatic bandwidth is slightly inferior to Ref. [28], the quality of achromaticity is superior to Ref. [28]. In addition, we note that our metalens is the first reported polarization-independent and achromatic Huygens’ metalens in the microwave region.

**Table 1:** Comparison of the proposed metasurface with other representative works.

Ref #	Metalens type	Polarized	Design principle^a^	Structure layers	NA	3 dB bandwidth	Achromatic bandwidth	Focusing efficiency^b^
[24]	Chromatic	Dual	Huygens	3	0.57	8–12 GHz (28 %)	N/A	61.2 %
[25]	Chromatic	Single	Reflection	2	0.51	5.5–6.5 GHz (16.7 %)	N/A	70 %
[26]	Chromatic	Single	Reflection	2	0.98	Single frequency at 22.4 GHz	N/A	48 %
[27]	Achromatic	Single	Huygens	3	0.28	Not given	8.5–9.5 GHz (11 %)	70 %
[28]	Achromatic	Single	FSS	6	0.67	Not given	9.8–12.2 GHz (21.8 %)	78.9 %
Our work	Achromatic	Dual	Huygens	3	0.64	21–30 GHz (30.7 %)	22–26 GHz (16.7 %)	72 %

^a^FSS means frequency selective surface. ^b^Refs. [24–26] use the maximum focusing efficiency, Refs. [27, 28] and our work use the average focusing efficiency.

We further compare our work to previous achromatic metalenses in terms of polarization, NA, focusing efficiency, structural complexity and lens thickness. Compared to the two achromatic metalenses [27, 28], our achromatic metalens is dual-polarized and works at a higher frequency. The NA and achromatic focusing bandwidth are both improved from Ref. [27] due to the use of multiresonant structures. Our achromatic metalens has a NA and focusing efficiency comparable to Ref. [28], but we have dramatically reduced the number of layers (from 5 to 2) and hence the metalens thickness (from 0.87*λ*_*c*_ to 0.13*λ*_*c*_). A thinner lens reduces the fabrication cost, is better for integration, and has better potential for handling wide-angle incidence.

## 4 Conclusions

In this paper, we have reported a dual-polarized achromatic Huygens’ metalens with large NA and effective focusing performance built using a compact and optimized structure. Compared to the earlier Huygens metalenses and achromatic metalens based on FSSs, we have improved the phase dispersion range with a multi-resonant unit cell, and achieved flexible control of the transmission phase and delay by tuning these resonances with only three metallic pattern layers. Simulation and measurements show that our metalens can achieve excellent achromatic focusing with a fractional bandwidth of 16.7 % over the frequency range of 22–26 GHz. Over the entire bandwidth, the measured focal shift is reduced by 89 % compared to a theoretical chromatic lens. Compared to the previous microwave achromatic metalens [21, 27, 28], our Huygens’ metalens boasts (i) polarization independence, (ii) a deep-wavelength thickness of about 0.13*λ*_*c*_, (iii) a large NA (0.64), a high focusing efficiency (72 % average) and a diffraction-limited spot size (Strehl ratio > 0.8), and (iv) a broad working bandwidth. Additionally, the achromatic metalens design methodology can be straightforwardly extended to higher frequencies. These advantages make this achromatic metalens an attractive candidate for microwave imaging, sensing and communication applications.

## Supplementary Material

Supplementary Material Details
