# Supplementary material for: Polarization-independent achromatic Huygens’ metalens with large numerical aperture and broad bandwidth
Source: Nanophotonics. 2023 Aug 18;12(18):3633–44. doi: 10.1515/nanoph-2023-0331 (PMC11501944; doi:10.1515/nanoph-2023-0331)
Supplement: Supplementary file 1 — Supplementary Material Details [file j_nanoph-2023-0331_suppl_001.pdf]

Supplementary

Xiaoluo He, Chu Qi, Sheng Lei, and A. M. H. Wong*

Polarization-independent achromatic Huygens' metalens with large numerical aperture and broad bandwidth

https://doi.org/10.1515/sample-YYYY-XXXX
Received Month DD, YYYY; revised Month DD, YYYY; accepted Month DD, YYYY

1 Supplementary Note S1. Analysis of three EM resonances

As shown in the main text, there are one magnetic and two electric resonances can be generated by the designed unit cell. The antisymmetric current of the outer layers generates the magnetic resonance, while two electric resonances are generated by the corner strips of outer layers and pairs of strips of the middle layer, respectively. We simulate the unit cell with only one variable each time to show how the design parameters affect these resonances. After that, we calculate the imaginary part of normalized electric admittance Y and magnetic impedance Z according to the Eq. (2) of the main text. The results are given in Figure S1 and S2.

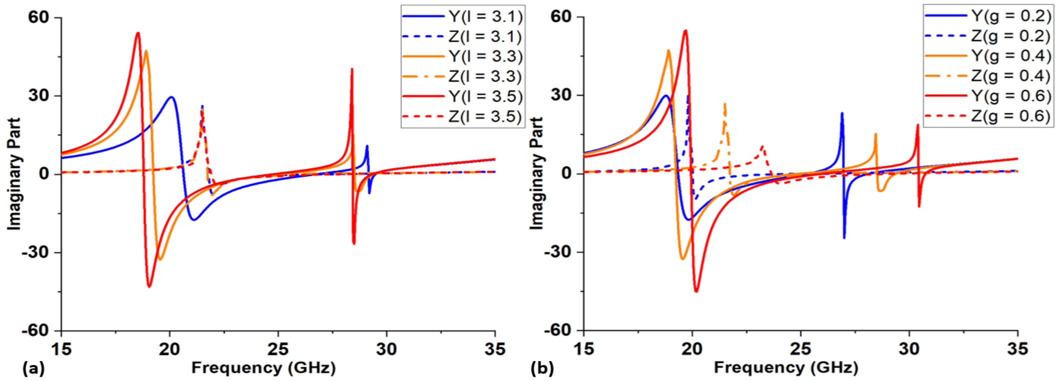

Supplementary Figure S1: The imaginary part of normalized electric admittance Y and magnetic impedance Z with (a) outer layers are fixed (g is fixed, l is the variable). (b) the middle layer is fixed (l is fixed, g is the variable)

Figure S1 gives the imaginary part of the normalized electric admittance and magnetic impedance with different values of $l's$ and $g's$. We find that when the middle layer changes, the magnetic response is almost unchanged, while the magnetic resonance position varies with the change of g. This is because the magnetic resonance is only generated from the antisymmetric surface currents between outer layers.

The first electric resonance is mainly determined by the surface currents of the middle layer as shown in Figure 4(c) of the main text (Page 6), but is also preturbed by coupled currents among all three layers.

***Corresponding author: A. M. H. Wong,** City University of Hong Kong, State Key Laboratory of Terahertz and Millimeter Waves, Department of Electrical Engineering, Hong Kong, China, e-mail: alex.mh.wong@cityu.edu.hk
Xiaoluo He, Chu Qi, Sheng Lei, City University of Hong Kong, State Key Laboratory of Terahertz and Millimeter Waves, Department of Electrical Engineering, Hong Kong, China, e-mail: xiaoluohe2-c@my.cityu.edu.hk, chuqi2-c@my.cityu.edu.hk, shenglei2-c@my.cityu.edu.hk

Therefore, the first electric resonance changes with both variables (g and l), but is mainly affected by the variable of the middle layer (l).

The second electric resonance is contributed by the symmetric surface currents of the outer layers, as shown in Figure 4(b) of the main text. Like the first electric resonance, the second electric resonance is also perturbed by the coupling terms among all three layers. Therefore, the second electric resonance shows great changes with the variation of g, and also changes slightly with the variation of l.

To further evaluate which parameters affect the second electric resonance, we add another variable here: the width of the corner strips of outer layers (w) (In our main text, w is fixed to 0.2mm for all unit cells), as shown in Figure S2(a). Then we calculate the imaginary part of Y and Z with a fixed g but different $w's$, as shown in Figure S2(b). We find the magnetic resonance position is unshifted, while the electric resonance shifts to higher frequency with the increase of w. Figure S1(b) and Figure S2(b) show that the second electrical resonance is mainly affected by the length and width of four corner strips of the outer layers.

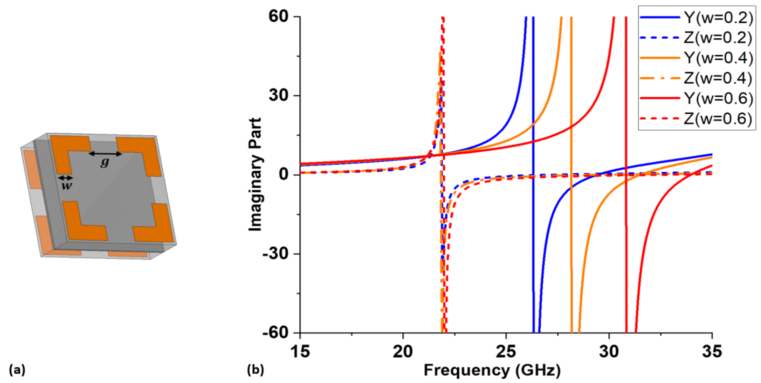

Supplementary Figure S2: (a) The schematic of outer layers. (b) Imaginary part of Y and Z with the variation of w.

2 Supplementary Note S2. Cascaded transfer matrix

We use the ABCD matrix to obtain the transmission coefficient of three-metallic-layer Huygens' metasurface, whose equivalent circuit diagram is shown in Figure 3(a). The ABCD matrix can be used to calculate the S-parameter [1, 2]. More specifically, S-parameters can be expressed in terms of the sheet admittances (Y_{s1} and Y_{s2}) and the substrate's impedance (Z_{sub}), wavenumber (β) and the thickness (h).

$$\begin{pmatrix} A & B \\ C & D \end{pmatrix} = \left[\begin{pmatrix} 1 & 0 \\ Y_{s1} & 1 \end{pmatrix} \begin{pmatrix} cos(\beta h) & jZ_{sub}sin(\beta h) \\ \frac{jsin(\beta h)}{Z_{sub}} & cos(\beta h) \end{pmatrix} \begin{pmatrix} 1 & 0 \\ Y_{s2} & 1 \end{pmatrix} \begin{pmatrix} cos(\beta h) & jZ_{sub}sin(\beta h) \\ \frac{jsin(\beta h)}{Z_{sub}} & cos(\beta h) \end{pmatrix} \begin{pmatrix} 1 & 0 \\ Y_{s1} & 1 \end{pmatrix} \right] \tag{1}$$

$$\begin{pmatrix} S_{11} & S_{12} \\ S_{21} & S_{22} \end{pmatrix} = \begin{pmatrix} \frac{\frac{B}{Z_{sub}} - CZ_{sub}}{2A + \frac{B}{Z_{sub}} + CZ_{sub}} & \frac{2}{2A + \frac{B}{Z_{sub}} + CZ_{sub}} \\ \frac{2}{2A + \frac{B}{Z_{sub}} + CZ_{sub}} & \frac{\frac{B}{Z_{sub}} - CZ_{sub}}{2A + \frac{B}{Z_{sub}} + CZ_{sub}} \end{pmatrix} \tag{2}$$

Thus, once geometric parameters of unit cell are specified, the S-parameters varies with the change of sheet admittances (Y_{s1} and Y_{s2}), as shown in Figure 3b-c of the main text. The sheet admittances are directly related to the three variables: the gap between corner strips of outer layers (g), the folded strip length of outer layers (l_1), and the length of four strips in the middle layer (l). The imaginary part of sheet admittances (Y_{s1} and Y_{s2}) can be calculated from the simulated transmission and reflection coefficients. The calculation method can be found in [1]. To show how the variables affect the admittance values, we give the calculated imaginary parts of admittance values with two cases, as shown in Figure S3. In the first case, we fix g to 0.4 mm and vary l from 3.1 mm to 3.5 mm. We find that Y_{s1} (the admittance of outer layers) stays almost unchanged, while Y_{s2} (the admittance of the middle layer) increases with l. Y_{s1} remains stable because it is mainly determined by the magnetic response, and the middle layer has no influence in the magnetic response. On the other hand, Y_{s2} changes with l because it is determined by the electric response. For the second case, we fix l to 3.3 mm, while g varies from 0.2 mm to 0.6 mm. We find that both Y_{s1} and Y_{s2} change with the changes of g, as the magnetic response and electric response are both related to the outer layers. Therefore, to obtain the desired sheet admittance efficiently, we first tune the gap g of the outer layers to get the desired Y_{s1}, then tune the length of the strips in the middle layer l to get the desired Y_{s2}.

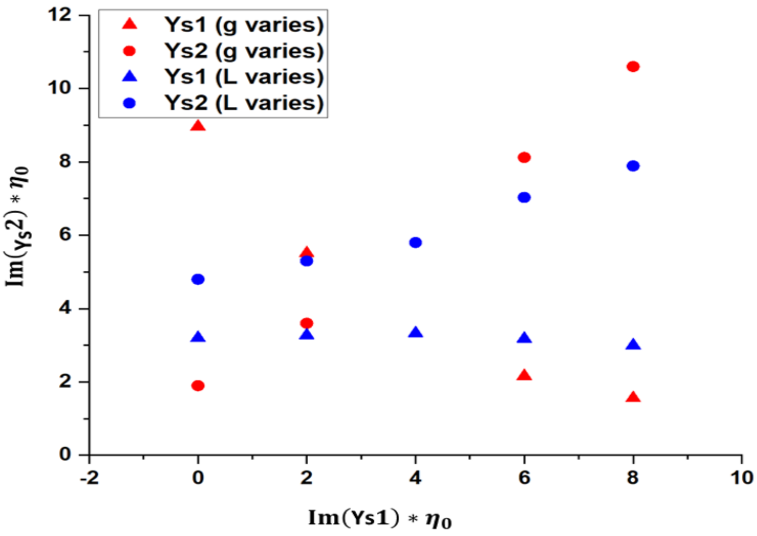

Supplementary Figure S3: The sheet admittances Y_{s1} and Y_{s2}) with different geometric parameters.

3 Supplementary Note S3. Imaginary parts of normalized Y_{es} and Z_{ms} of all unit cells.

To further illustrate the relationship between the group delay and the electromagnetic resonances, we plot the imaginary parts of normalized admittances and impedances of all unit cells, as shown in Figure S4. The transmission properties of these unit cells as a function of frequency are plotted in Figure 7 of the main text. We can find unit cells with large group delays have more resonances near the frequency range of interest (22 - 26 GHz). In contrast, the resonance frequencies of unit cells with small group delay are far from the frequency range of interest. The resonances frequency for the complete unit cell (which is the combination of Y and Z) occurs at which $\mathrm{Im}\{Y\} = \mathrm{Im}\{Z\}$. We use red circles to mark the resonance frequencies of each unit cell as shown in Figure S4, and these resonance frequencies correspond to the frequencies of maximum transmissions.

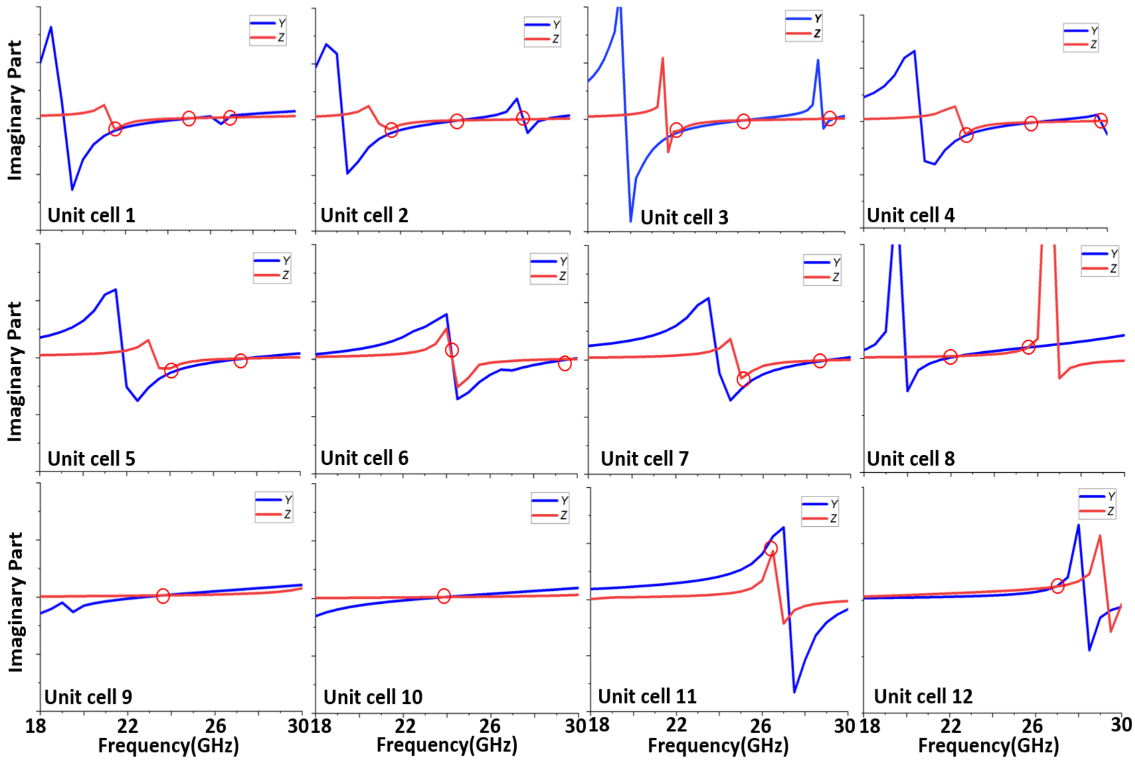

Supplementary Figure S4: The imaginary parts of Y and Z of unit cells (Red circles: the resonances of the complete unit cell.)

4 Supplementary Note S4. Comparison of two achromatic metalenses with different NAs

Figure S5 compares the required phase distributions and relative group delays of two achromatic metalenses with NAs of 0.32 (Figure S5 (a) and (c)) and 0.64 (Figure S5 (b) and (d)) at a frequency of 24 GHz. Both achromatic metalenses have the same radius (66 mm) but have different focal lengths. As shown, the achromatic metalens with an NA of 0.32 needs a phase range of about 360° at 24 GHz and a group delay tuning range of about 35 ps. However, the achromatic metalens with a larger NA of 0.64 requires a phase range of about 720° at 24 GHz and a group delay tuning range of about 80 ps.

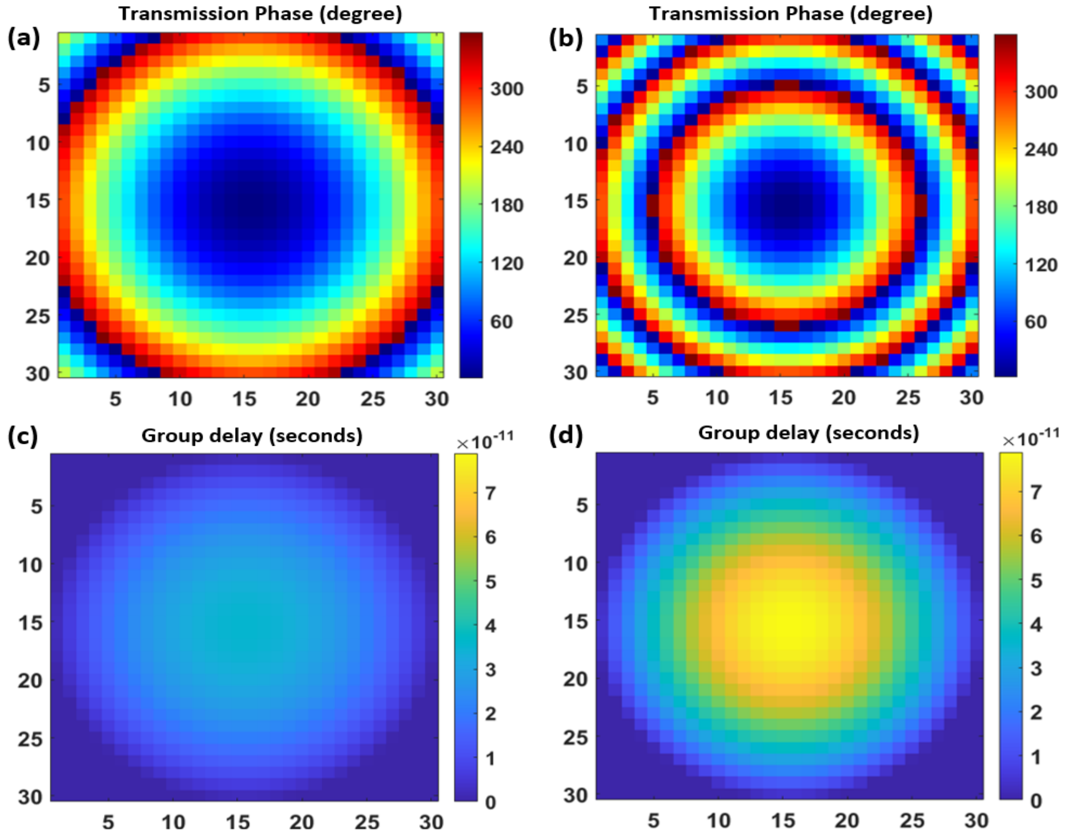

Supplementary Figure S5: The comparison of achromatic metalenses with different NAs. (a) The required phase distribution of achromatic metalens with NA= 0.32. (b) The required phase distribution of achromatic metalens with NA= 0.64. (c) The required group delay distribution of achromatic metalens with NA= 0.32. (d) The required relative group delay distribution of achromatic metalens with NA= 0.64

5 Supplementary Note S5. Design parameters of the meta-atoms

Supplementary Table S1: The geometrical parameters and corresponding group delay of unit cells

Number of Unit Cell	Group Delay (ps)	g (mm)	l (mm)	l_1 (mm)
1	108	1.1	3.4	0.8
2	106	0.3	3.4	0.2
3	105	0.4	3.3	0.2
4	104	0.5	3.1	0.2
5	93	0.6	2.9	0.2
6	80	0.7	2.6	0.2
7	70	1.2	2.5	0.5
8	65	0.1	3.7	1.5
9	58	0.2	3.7	1.1
10	53	0.1	3.8	0.9
11	45	0.9	1.8	0.2
12	30	1.5	3.8	0.2

6 Supplementary Note S6. Oblique incidence performance of the achromatic metalens

Apart from the chromatic aberration, the field of view is another important parameter to evaluate the performance of metalens. Here, we plot the focusing performance of our achromatic metalens under 15° and 30° incidences, as shown in Figure S6 and Figure S7. We observe that the chromatic correction still works upon the oblique incidence of 30° across the frequency range of interest (22 GHz to 26 GHz). Compared to the normal incidence, the focal lengths is shifted to 77 mm and 70 mm for 15° incidence and 30° incidence, respectively. The focal length can be expressed as:

$$F_{\theta} = F_{0^{\circ}} \cos(\theta) \tag{3}$$

Besides, the focal spots have a low sidelobe level, and average FWHMs of spot size under 15° and 30° incidences are about 1.19 and 1.33 times the diffraction-limited spot size over most of the working bandwidth. (except 30° incidence of 26 GHz). This shows that our achromatic lens can still work effectively upon the oblique incidence up to 30°.

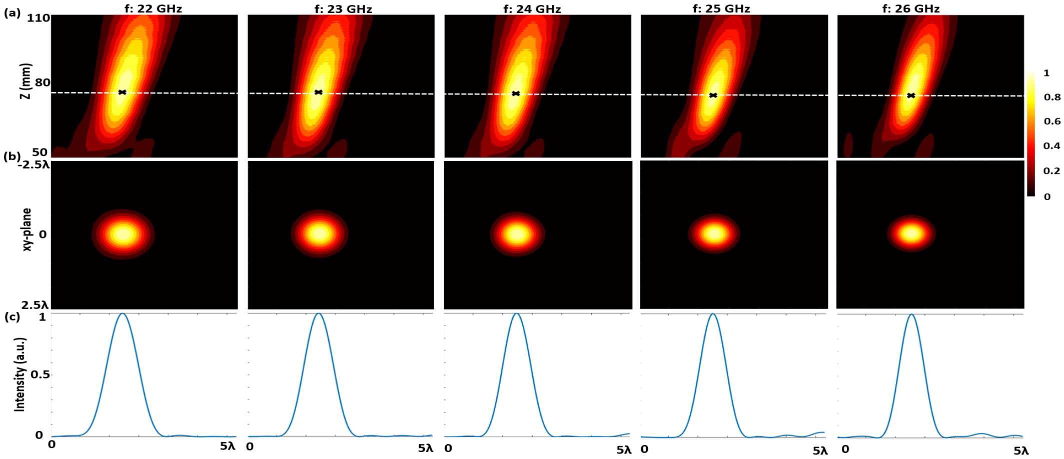

Supplementary Figure S6: The focusing performance of the achromatic metalens under 15° incidence

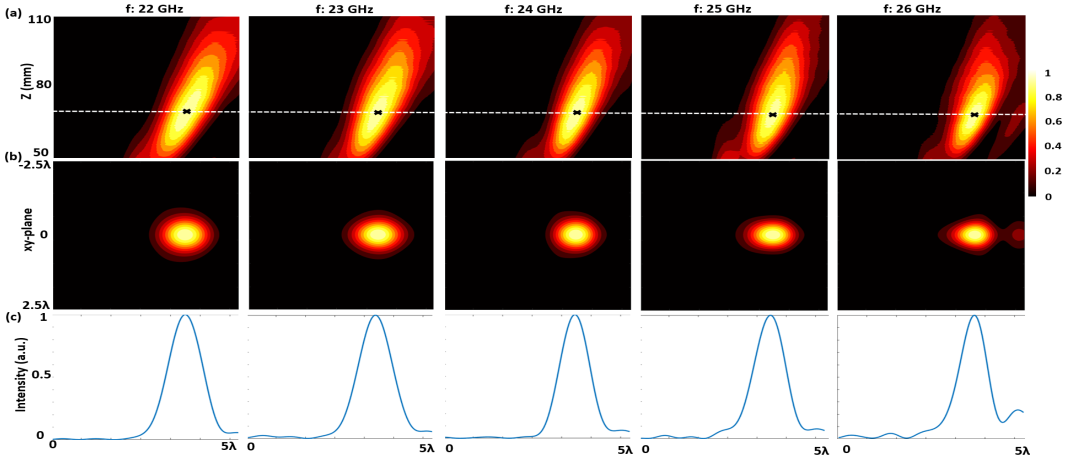

Supplementary Figure S7: The focusing performance of the achromatic metalens under 30° incidence

7 Supplementary Note S7. 3dB focusing bandwidth

In Figure S8, we give the simulated intensity distribution along the propagation plane, normalized by the peak intensity over the entire bandwidth. While the metalens achieves optical achromatic performance from 22 GHz to 26 GHz, it achieves efficient focusing the entire frequency range from 20 - 31 GHz. Figure S9 plots the metalens' normalized focusing efficiency (i.e. the focusing efficiency as a function of frequency divided by the peak focusing efficiency across the entire considered bandwidth). It can be seen that the 3dB bandwidth stretches from 21-30 GHz in the simulated results and from 22-30 GHz in the experimental results. The measured fractional bandwidth of 30.7% surpasses most previous single and dual polarized chromatic and achromatic microwave metalenses.

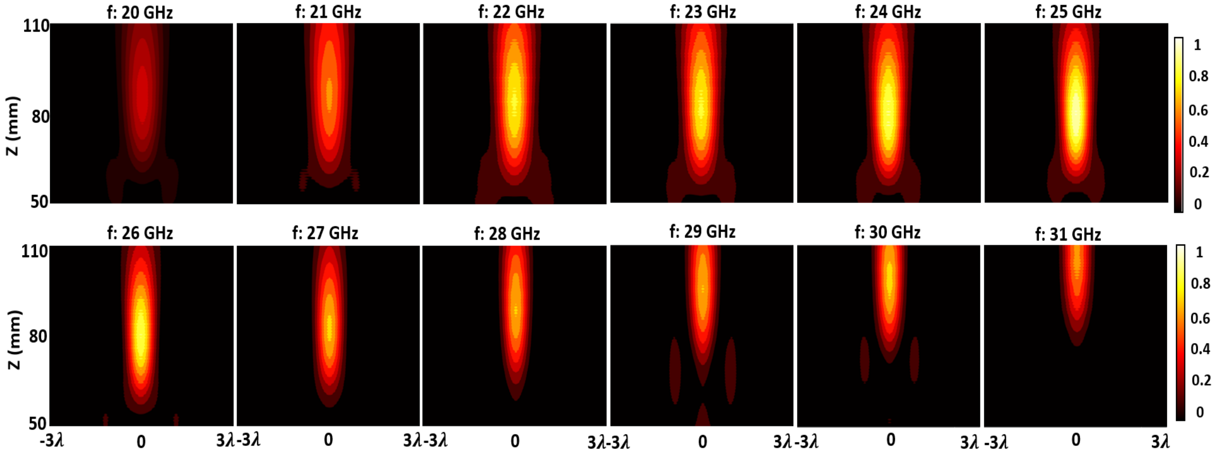

Supplementary Figure S8: Simulated intensity distribution of the achromatic metalens.

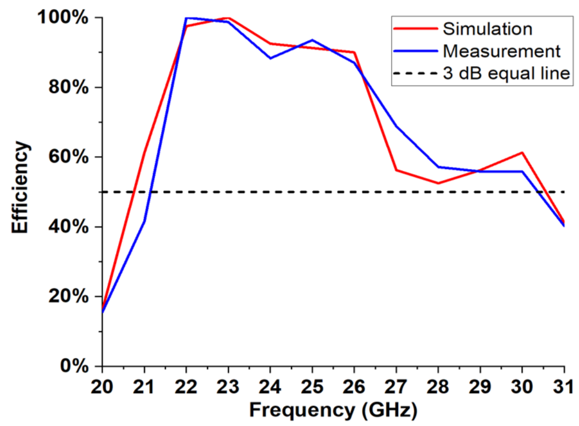

Supplementary Figure S9: Normalized focusing efficiency of the achromatic metalens.

References

[1] C. Pfeiffer and A. Grbic, "Millimeter-wave transmitarrays for wavefront and polarization control," *IEEE Trans. Microw. Theory Tech.*, vol. 61, no. 12, 2013, pp. 4407-4417. https://doi.org/10.1109/TMTT.2013.2287173

[2] D. M. Pozar, "Microwave engineering," John wiley & sons, Berlin, 2011.
